# Cruelties of the Voting System

**Published:** 1903-09-19

**Authors:** 


### CRUELTIES OF THE VOTING SYSTEM.

### WHERE ARE THE COMMITTEES OF MANAGEMENT?

Fanny Sargeaunt Durrant, aged 60, governess, suffering from internal disease and debility, came up four times for election as a pensioner of a hospital for incurables, and on the fifth occasion when the governors voted for her once more the voting paper was returned with the word "dead" written across the vote. The added suffering and misery of the waiting and the doubt, the alternating periods of hope deferred and despair at failure, and the ultimate result, death, before any relief was given, should appeal to the sympathies of every thinking man and woman among the governors, and make them determine that the present system of electing pensioners should be modified in favour of one which shall secure at any rate that so soon as a case is declared by the committee to be eligible for admission to a pension that pension shall be forthcoming for certain within six months at the outside. How many hundreds of cases must there be equally pathetic, and probably even more condemnatory of a system of election which adds all the cruelties referred to to the many difficulties and impediments which must necessarily interpose between the chronic incurable and the monetary or institutional relief which each case urgently needs. The more needy in a pecuniary sense the circumstances of a particular sufferer, the greater appears to the difficulty of securing the relief which the institutions were established to offer. In another case which came under our notice quite recently an unfortunate seaman in his old age wrote for a vote for the Royal Alfred Institution, being in support of his sixteenth application to the governors for election. We wonder what the committee of an institution can be about which permits a system so cruel and inefficient as this to continue for a moment beyond the necessary time required to reform and amend it.

